# Transfusion service knowledge and immunohaematological practices related to sickle cell disease and thalassemia

**DOI:** 10.1111/tme.12580

**Published:** 2019-02-10

**Authors:** R. M. Fasano, J. Branscomb, P. A. Lane, C. D. Josephson, A. B. Snyder, J. R. Eckman

**Affiliations:** ^1^ Center for Transfusion and Cellular Therapies, Department of Pathology and Laboratory Medicine Emory University School of Medicine Atlanta Georgia USA; ^2^ Georgia Health Policy Center, Andrew Young School of Policy Studies Georgia State University Atlanta Georgia USA; ^3^ Aflac Cancer and Blood Disorders Center, Children's Healthcare of Atlanta, Department of Pediatrics and Hematology/Oncology Emory University School of Medicine Atlanta Georgia USA; ^4^ Department of Hematology & Oncology Emory University School of Medicine Atlanta Georgia USA

**Keywords:** red cell transfusion, sickle cell disease, thalassemia major, transfusion practices

## Abstract

**Objectives:**

To assess current knowledge of National Heart, Lung and Blood Institutes (NHLBI) and Thalassemia International Federation (TIF) recommendations, blood banking practices and perceived challenges among transfusion services in the management of patients with haemoglobinopathies.

**Background:**

Previous reports have demonstrated variations in transfusion practices for sickle cell disease (SCD) and thalassemia patients. Recently, NHLBI/TIF have provided transfusion recommendations for patients with haemoglobinopathies.

**Methods:**

A cross‐sectional survey was conducted of transfusion services from the state of Georgia previously identified as having SCD/thalassemia populations. The survey assessed transfusion service practices in pre‐transfusion testing and blood product selection; awareness/implementation of NHLBI/TIF transfusion‐based recommendations and perceived challenges in transfusing haemoglobinopathy patients.

**Results:**

Responses were received from 35 of 49 (71%) institutions. Only institutions indicating transfusing SCD or thalassemia patients (32) were included in analysis. Seventy‐one percent of non‐sickle cell treatment centres (SCTCs) and 20% of non‐thalassemia treatment centres follow NHLBI and TIF recommendations to perform a red blood cell phenotype beyond ABO/Rh(D) and provide Rh and Kell prophylactically matched units for SCD and thalassemia patients, respectively. Forty percent of institutions (33% of non‐SCTCs) employ RBC genotyping to evaluate the red cell phenotype for SCD patients. Over 77% of institutions do not utilise a reliable method to identify SCD patients prior to transfusion, such as a required question/answer field on type/screen or crossmatch orders.

**Conclusion:**

Many healthcare systems' transfusion practices for haemoglobinopathy patients are discordant with NHLBI/TIF recommendations. Efforts are needed to increase awareness and implementation of current recommendations among all transfusion services seeing these patients.

Red blood cell (RBC) transfusions are a key component of the comprehensive management of patients with sickle cell disease (SCD) and thalassemia; however, they are not devoid of risk. Advances in transfusion practices and clinical care have led to improved safety of transfusion therapy for patients with haemoglobinopathies with regard to transmission of blood‐borne infectious agents, RBC alloimmunisation, transfusional iron overload and suspected transfusion reactions. However, transfusion practices vary among providers and transfusion services for these patients.

Previous surveys of blood bank medical directors, laboratory supervisors and providers at Comprehensive Sickle Cell and Thalassemia Treatment Centres in the United States have demonstrated a lack of consistency on transfusion practices for SCD and thalassemia (Afenyi‐Annan *et al.,* 2007; Goss *et al.,* 2014; Lal *et al.,* 2018). Over 10 years ago, results of a data set from a College of American Pathologists (CAP) Proficiency Testing Survey assessing over 1100 transfusion service practices for non‐alloimmunised SCD patients demonstrated that less than 30% of North American hospital transfusion service laboratories determined SCD patients' baseline RBC serologic phenotype beyond ABO and Rh(D) and issued RBC units that were matched for Rh (C/c, E/e) and Kell antigens (Osby & Shulman, 2005). More recently, Dunbar *et al*. (2012) published results of a survey aimed at characterising transfusion practices of SCD patients among haematology/oncology providers within the state of Florida. This survey included adult and paediatric haematologists/oncologists, of which non‐academic and adult‐oriented clinicians represented a large proportion of the respondents. The results again indicated a wide range of transfusion practices, and the majority of respondents did not routinely request phenotypically matched RBCs for SCD patients until the patient demonstrated a new antibody. When compared to practices at comprehensive sickle cell centres where more than 90% routinely provided Rh (C/c, E/e)‐matched and Kell antigen‐matched RBCs, this highlighted significant inconsistencies in practices between comprehensive sickle cell centres and community‐based healthcare systems. Furthermore, it has been previously shown that only half of the thalassemia treatment centres provide Rh (C/c, E/e)‐matched and Kell‐matched RBCs for transfusions in thalassemia patients (Goss *et al.,* 2014), and data on transfusion practices at non‐thalassemia treatment centres, community‐based institutions, are currently lacking.

The National Heart, Lung and Blood Institutes (NHLBI) and the Thalassemia International Federation (TIF) published expert panel recommendations for SCD and guidelines for transfusion dependent thalassemia (TDT), respectively, in 2014. These include comprehensive recommendations for transfusion therapy in patients with SCD and TDT, and advocate for all such patients to receive prophylactic Rh (C/c, E/e)‐matched and Kell‐matched RBCs to reduce the risk of alloimmunisation (Trompeter & Cohen, 2014; Yawn *et al.,* 2014). However, this practice is still not likely implemented universally, due to a lack of awareness of these recommendations or increased costs incurred, especially at community‐based institutions. Additionally, there are facets of transfusion service practices encompassing patient RBC serologic phenotyping (or genotyping), pre‐transfusion testing and blood product processing and selection that are not adequately addressed in NHLBI or TIF guidelines due to a lack of evidence of sufficient quality to guide clinical practice.

Therefore, we sought to survey hospital transfusion services throughout the state of Georgia to assess current knowledge of NHLBI and TIF evidence‐based recommendations, blood banking practices and perceived challenges in the management of patients with these haemoglobin disorders. We hypothesised that blood banking practices at non‐sickle cell and thalassemia treatment centres and community‐based institutions do not fully adhere to NHLBI/TIF recommendations.

## METHODS

The study was supported by a Centres of Disease Control and Prevention (CDC) cooperative agreement (DD14‐1406) to reduce transfusion‐related complications for people with haemoglobin disorders. It was approved by Georgia State University's institutional review board.

### *Study population*

The study targeted hospital blood bank medical directors, laboratory managers, supervisors and medical technicians at institutions where Georgia residents with haemoglobinopathies might seek treatment. Participants were solely recruited at an annual regional meeting of Southeastern Area Blood Bankers (SEABB), with additional, targeted, telephone recruitment to reach Georgia hospitals that collectively provided 90% of all SCD/thalassemia transfusions in the years 2004 through 2008, as identified through prior surveillance (Hulihan *et al.,* 2015). We sent email invitations containing an open link to the self‐administered online survey to subjects in two waves. The first wave was distributed in October 2015 to 121 email addresses from the SEABB meeting recruitment. Of these, 20 were undelivered. The second wave was distributed in June 2016 to nine addresses from the telephone recruitment, all of which were delivered.

Institutions were classified as ‘Sickle Cell Treatment Centers (SCTCs)’ based on being either an NHLBI historically designated Comprehensive Sickle Cell Centre, or currently receiving state funding for comprehensive sickle cell services including newborn screening follow up. Additionally, institutions were classified as ‘Thalassemia Treatment Centers (TTCs)’ based on being previously funded by CDC as part of the Thalassemia Clinical Research Network.

### *Survey development*

A team that included experts in paediatric (P. A. L., R. M. F., C. D. J.) and adult (J. R. E.) haematology, transfusion medicine (R. M. F., C. D. J.) and qualitative research (J. B.) developed the questions and designed the survey for the web‐based software (Qualtrics version 10/2015, Provo, UT, USA). The instrument was pilot‐tested by other haematologists and blood bank personnel from outside of the study's geographic scope. The instrument is included as a supplemental figure.

The survey questions addressed here covered respondent and institution characteristics, and current practices in transfusion to haemoglobinopathy patients. Additional questions on training needs and preferred learning channels are not reported here but were asked to inform follow‐up activities.

### *Data analysis*

Results were exported from Qualtrics to Microsoft Excel (Microsoft Office Professional Plus 2013, version 15.0.4981.1000) for analysis. Descriptive statistics for categorical variables are reported using proportions. Open‐ended text responses were grouped thematically using inductive analysis.

## RESULTS

A total of 49 institutions were recruited for the survey, with 35 responding (71%). Multiple responses were received from staff at six institutions. In these cases, the response of the most senior or highly trained individual, by self‐report, was retained for this analysis. Three responding institutions reported providing no SCD or TDT transfusions and were also omitted from this analysis. Included institutions represented 23 Georgia counties and 3 neighbouring states (Tennessee, Alabama and South Carolina). Three institutions were categorised as SCTCs, and one of these also as a TTC. Additional descriptive data for the sample are provided in Table S1, Supporting Information.

### *Institution characteristics*

**Figure 1:**
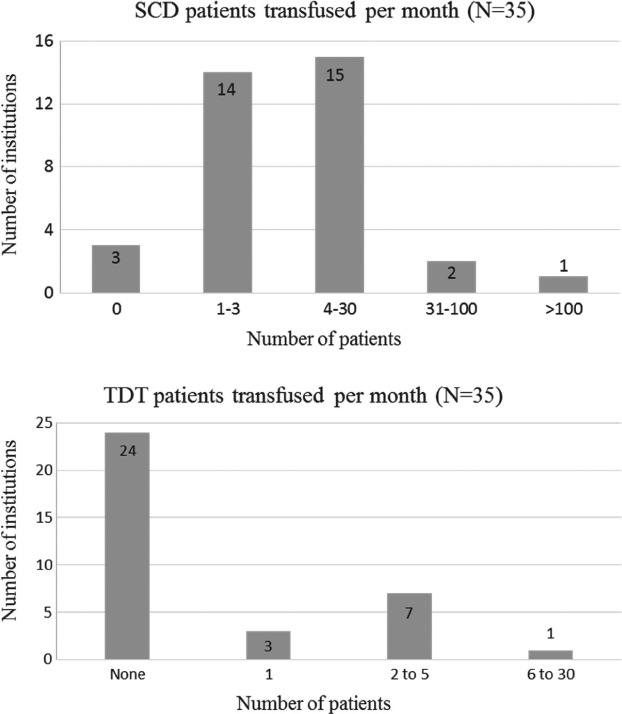
Number of SCD patients (a) and TDT patients (b) transfused per month from 35 institutions surveyed. Responses regarding SCD and TDT transfusion practices are reported for the 32 and 11 institutions, respectively, who stated they transfuse these patients. Note: although distributed throughout almost all areas of the state, the SCD population is overwhelmingly concentrated in the state's largest urban area, where one comprehensive centre serves a high volume of patients needing transfusion.

All institutions reported using blood from external collection facilities. Only one reported on‐site collection of any of the RBC units issued; and those were estimated to constitute less than 10% of all transfused units. Of the 32 institutions reported providing transfusions for SCD patients, 10 (31%) provided manual exchange transfusions and 13 (41%) provided automated RBC exchange transfusions. The major prescribers of RBC transfusion for SCD/TDT patients included adult haematologists/oncologists, internists, hospitalists, paediatric haematologists/oncologists and emergency medicine physicians. Reported frequency of transfusing SCD and TDT patients is shown in Fig. 1. Of 11 included responding institutions that reported transfusing thalassemia patients, only one (the TTC) reported transfusing six or more thalassemia patients per month. Eleven institutions (34%) transfused only adult SCD patients; another nine (28%) report that >90% of their transfused SCD patients are adults and four (13%) transfuse predominantly (>90%) paediatric SCD patients. Fourteen institutions (44%) provided a chronic transfusion therapy programme for SCD patients. Ten institutions (40% of the 25 answering this question) stated that the need for red cell exchange (erythrocytaphaeresis) would trigger a request for physical transfer of a patient to a specialised apheresis centre. Development of complicated RBC alloantibodies, autoantibodies or need for extended match units (beyond C/c, E/e, K) was additional reasons for referral.

#### Blood bank practices

RBC component processing practices for SCD and TDT patients are illustrated in Table 1. Leukoreduced RBCs are given almost universally to SCD and TDT patients; all medical centres provided sickle cell trait–negative RBCs to SCD patients, whereas only half of the responding institutions provided sickle cell trait–negative RBCs to TDT patients. Irradiated RBCs were rarely used for SCD or TDT patients (approximately 10% of institutions), and under 20% of institutions had unit age restrictions for either SCD or TDT patients. Similarly, few imposed storage solution restrictions for either SCD or TDT patients.

**Table 1:** Required RBC component processing and matching for transfusion to SCD and TDT patients

	SCD (*N* = 32)	TDT (*N* = 11)
Leukoreduced	30 (94%)	10^1^ (100%)
Irradiated	3 (9·4%)	1^1^ (10%)
Negative for sickle cell trait	32 (100%)	5^1^ (50%)
Unit age restriction	3^2^ (9·4%)	2^1^^,^ 3 (20%)
Storage solution^1^^,^ 4	5^5^ (16%)	
Prophylactic matching beyond ABO/Rh(D)	23^1^ (74%)	3 (27%)

1 One missing response.

2 1: <14 days; 1: <21 days; 1: freshest available.

3 1: <14 days; 1: <21 days.

4 This question not asked separately with respect to SCD/TDT patients.

5 4: AS‐1, AS‐3 (12%); 1: CPDA (3·1%).

#### RBC phenotyping and antigen matching

**Figure 2:**
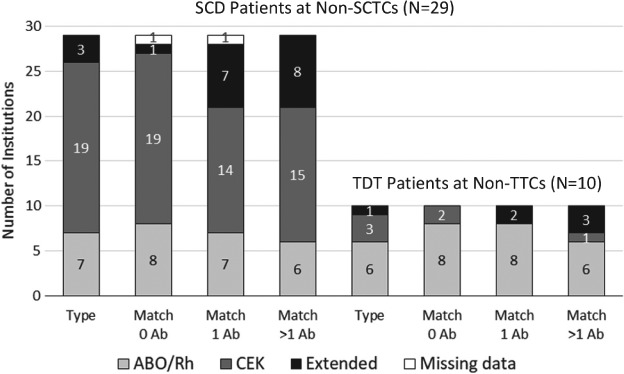
Phenotyping and antigen matching for transfusion in SCD and TDT patients at non‐SCTCs and non‐TCCs. For patients with known history of alloantibodies, all institutions report providing RBC units matched for those known antibodies with various degrees of additional antigen matching. One non‐SCTC skipped two of the four questions in this series. Note: Prophylactic matching for Rh C/c, E/e and K in addition to ABO/Rh(D) was reported by all three SCTCs and the TCC; extended matching was reported for all three SCTCs and the TCC for patients with one or more RBC antibodies (data not shown in figure). Ab = alloantibodies; type = phenotype.

Routine blood group antigen phenotyping practices of both SCD and TDT patients varied among non‐SCTCs/non‐TTCs. Although the TTC and two of three SCTCs perform extended phenotyping (beyond ABO/Rh(D), C/c, E/e and K) for non‐alloimmunised TDT and SCD patients, only 10% of non‐TTCs/non‐SCTCs perform upfront extended phenotyping for all TDT/SCD patients. Similarly, the types of RBC products routinely provided to SCD and TDT patients differed among institutions. Prophylactic matching for Rh C/c, E/e and K in addition to ABO/Rh(D) was reported by all SCTCs and TCCs; but only 20 (71%) non‐SCTCs and two (20%) non‐TTCs follow this practice (Fig. 2). Furthermore, whereas all SCTCs and TTCs reported providing extended matched RBCs once alloimmunisation occurred, several non‐SCTCs/TTCs do not.

#### RBC genotyping

Out of 27 non‐SCTCs that answered the survey question on the use of RBC genotyping, 18 (67%) reported that they do not employ this method to evaluate the red cell phenotype for SCD patients. One of the three SCTCs performs RBC genotyping for all SCD patients, and two SCTCs and nine (33%) non‐SCTCs utilise RBC genotyping on a case by case basis – most commonly for patients with complicated antibody reactivity and/or to determine whether an autoantibody vs an alloantibody is present (e.g. anti‐e in an e+ patient because of an Rh variant haplotype). Other indications included: new SCD patients without appropriate transfusion or antibody history, or if the immunohaematology reference laboratory (IRL) recommends molecular testing based on a complex workup.

### *Transfusion challenges for SCD/TDT patients*

The main challenges identified in the transfusion management of SCD and TDT patients (*N* = 30, incorporating responses from multiple staff at the same institution) included: obtaining a reliable and complete transfusion and antibody history across multiple hospital systems (*n* = 8, 26·7%), lack of standardised procedures for notifying transfusion services that a patient has either SCD or TDT (*n* = 6, 20%); a lack of standard policies/procedures (within a health system or across hospitals) for RBC phenotyping and product matching (*n* = 7, 23·3%) and securing appropriately matched RBC units for alloimmunised patients (*n* = 11, 36·7%).

**Figure 3:**
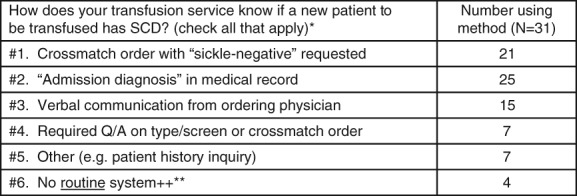
Response combinations for how transfusion services at SCTCs (*n* = 3) and non‐SCTCs (*n* = 28) know if a new patient to be transfused has SCD (*n*_total_ = 31). *Multiple answers were given for 24 institutions, the most common combination being: ‘#1. and #2.’ (n = 5); and #1, #2 and #3 (n = 5). **Respondents that indicated that they have ‘#6. No routine system’ for identifying that a patient to be transfused has SCD (all non‐SCTCs) also marked other mechanisms applied (*n* = 4), none of which included ‘#4. Required Q/A on type/screen and crossmatch’ order.

When transfusion services were asked how they are notified that a patient has SCD (Fig. 3), most responded that they utilise the ‘admission diagnosis’ identified in the medical record, along with other measures including: identification through a crossmatch order with ‘sickle‐negative’ restriction requested; a required question/answer field on the type/screen or crossmatch order asking if the patient has SCD and a verbal communication from the ordering physician. Seven (22·6%) institutions utilise (with or without other measures) a required question field on the type/screen or crossmatch order asking if the patient has SCD. Seven institutions (22·6%) reported that they have either no routine system or depend solely on verbal communication from the ordering physician for informing the transfusion service that a new patient has SCD.

With regard to preventing delayed haemolytic transfusion reactions (DHTRs), 10 (32·3%) institutions indicated they have no routine system in place for identifying SCD patients who may have been transfused elsewhere. Nine (29%) depend on the patients' clinical provider to obtain a transfusion history to identify whether transfusions have occurred at other institutions (clinical provider‐initiated). If there is a history of multisite transfusions, the provider notifies the transfusion service to contact the institution(s) where the patient was transfused to obtain antibody history prior to cross‐matching units. Fourteen (45·2%) contact the provider who orders a type/cross on a new SCD patient to obtain a transfusion history. A subgroup five (16·1%) institutions stated that their transfusion service contacts the provider ordering a type/cross on a SCD patient based on certain qualifications to obtain an interim transfusion history. These qualifications included if the direct antiglobulin test or antibody screen is positive, or if no RBC phenotype is on file for a given patient.

## DISCUSSION

The findings of this survey provide insights into the inconsistencies in transfusion practices of SCD and thalassemia patients in a geographic region densely populated with these patient populations. Similar to previously published reports (Afenyi‐Annan *et al.,* 2007), our results highlight adherence to NHLBI/TIF recommendations by SCD and Thalassemia treatment centres, but wide variation in transfusion practices among other healthcare systems. This may be due to unfamiliarity with these recommendations. Alternatively, this may represent a reluctance to adopt ‘cost‐adding’ NHLBI/TIF recommendations due to a lack of high‐quality evidence supporting that universal implementation of RBC antigen matching transfusion protocols significantly impacts patients' risks of haemolytic transfusion reactions (Fasano *et al.,* 2018). Compared to SCTCs that all indicated that baseline RBC phenotyping for at least Rh (C/c, E/e) and Kell antigens is performed and RBCs are provided, which are matched up front (prior to RBC alloimmunisation) for Rh (C/c, E/e) and K, over 25% of non‐SCTC institutions reported not to perform a RBC phenotype beyond ABO and Rh(D) or provide RBC units that are prophylactically matched for Rh (C/c, E/e) and Kell antigens in accordance with NHLBI recommendations (Yawn *et al.,* 2014). Although this is improved from a previous transfusion service survey conducted over a decade ago (in 2005) (Osby & Shulman, 2005), these results still highlight significant gaps in adherence to transfusion recommendations in non‐SCTC community‐based healthcare systems. Similarly, only 40% of non‐TTCs reported obtaining RBC phenotype beyond ABO and Rh(D) and 80% failed to provide RBC units prophylactically matched for Rh (C/c, E/e) and Kell antigens in accordance with TIF recommendations (Trompeter & Cohen, 2014). Interestingly, a subset of institutions described employing unit age and/or irradiation restrictions for SCD and TDT patients in the absence of any NHLBI/TIF recommendations to do so.

Despite prophylactic matching for Rh (C/c, E/e) and Kell antigens to prevent alloimmunisation, this phenomenon does occur and sometimes results in DHTRs, one of the most life‐threatening consequences of RBC alloimmunisation. This is especially the case with SCD patients because of their potential for bystander haemolysis of the patient's own erythrocytes and exacerbation of other sickle related complications. Whereas non‐SCD patients may have haemolysis of the transfused unit(s), patients with SCD may also have haemolysis of the transfused units in addition to their own endogenous sickle cell erythrocytes resulting in a post‐transfusion haemoglobin level that is significantly lower than the pre‐transfusion haemoglobin level, approximately 1 to 3 weeks after the inciting transfusion (Danaee *et al.,* 2015; Habibi *et al.,* 2016). Although DHTRs are often under recognised because of their tendency to mimic many sickle cell‐related manifestations (i.e. vaso‐occlusive crisis, acute chest syndrome), they occur in approximately 4·8 to 7·7% of adult SCD patients (rate: 3·5–4·2 per 100 episodic transfusions) (Vidler *et al.,* 2015; Narbey *et al.,* 2017) and are associated with a 6% mortality rate (Habibi *et al.,* 2016). Approximately 50% of adult SCD patients are alloimmunised despite prophylactic matching for Rh (C/c, E/e) and Kell antigens (Chou & Fasano, 2016). A high percentage of these RBC alloantibodies evanesce over time, and the disappearance varies with antigenic specificity (Tormey & Stack, 2009). Furthermore, multi‐site transfusion has also been well documented in SCD patients receiving multiple transfusions (Harm *et al.,* 2014; Unni *et al.,* 2014). Harm *et al*. documented that over 90% of alloimmunised SCD patients with evanesced antibodies were transfused at hospitals other than the hospital where the antibodies were detected, and that over 28% of these patients were transfused over 20 times in these hospitals after the antibodies have evanesced. Lastly, phenotypic matching may not prevent DHTR due to variant Rh antigens, which are relatively common among SCD patients. These circumstances all highlight a significant setup for DHTRs in the SCD population through re‐exposure to an evanesced alloantibody through an off‐site transfusion, and support the critical need for detailed and accurate inter‐institutional transfusion history sharing through regional and national transfusion registries in order to reduce the risk of DHTRs in this patient population. They also highlight the importance of federally funded cooperative projects such as the CDC (DD14‐1406) to reduce life‐threatening transfusion‐related complications for patients with haemoglobinopathies.

Our results highlight the need for quality initiatives to improve intra‐institutional and inter‐institutional communication for transfusion‐related information for detecting alloimmunised patients and preventing DHTRs. Despite the expanded use of electronic medical recording and ordering of blood products, many institutions surveyed have inadequate safeguards in place for determining patients at risk for adverse transfusion reactions such as RBC alloimmunisation, and, therefore, preventing DHTRs, many of which are severe and sometimes fatal. Only 22·6% of the institutions surveyed utilise a required question/answer field on the type/screen or crossmatch order asking whether the patient has SCD. The remaining transfusion services either perform a patient history inquiry themselves, or depend on the admitting diagnosis, crossmatch order with ‘sickle‐negative’ restriction requested or verbal communication from the ordering physician. In addition, a subset of the institutions surveyed states they have no routine system for identifying if a transfused patient has SCD (12·9%), and approximately a third state there is no routine system in place for identifying SCD patients who may have been transfused elsewhere, thereby limiting their ability to prevent DHTRs from occurring.

These survey results also provide insight into the potential need for expanding the role of advanced technologies in the transfusion management for patients with haemoglobinopathies, notably RBC genotyping and automated red cell exchange (for SCD patients), through increased education and training, or centralising their use in resource limited geographic areas. With regard to RBC genotyping, only one institution (a SCTC) reported to perform RBC genotyping to predict a RBC phenotype in all SCD (and TDT) patients; whereas 60% of transfusion services described not using RBC genotyping at all. This is despite the fact that automated DNA extraction and the ability to test large patient groups on high‐throughput molecular platforms are now readily accessible, and there currently is at least one molecular platform that is US food and drug administration approved as ‘test of record’, which means that the RBC phenotype determined by molecular testing does not require confirmation by serologic methods (Fasano *et al.,* 2017). Furthermore, more than one‐third of the centres indicated utilising this technology, through collaboration with immunohaematology reference laboratories that have access to higher‐fidelity genotyping assays, to aid in the evaluation of complicated antibodies, the most common scenario being to evaluate a SCD patient for an *RH* variant. This highlights the blood bank community's increased awareness that despite serologic matching for Rh antigens, SCD patients continue to form antibodies against blood group antigens within the Rh system because of the high prevalence of *RH* variants in this population (Chou *et al.,* 2013; Yee *et al.,* 2017).

In the survey, 40% of institutions noted that a need for automated red cell exchange (erythrocytaphaeresis) would result in a transfer to a centre with an apheresis service. This finding highlights the fact that acute erythrocytaphaeresis may be unavailable for many SCD patients with severe life‐threatening sickle‐related complications, and that chronic erythrocytaphaeresis may be under‐utilised as an iron‐sparing transfusion therapy for SCD patients in need of long‐term chronic transfusion therapy, despite considerable data showing its beneficial effects (Kim *et al.,* 1994; Adams *et al.,* 1996; Hilliard *et al.,* 1998; Singer *et al.,* 1999; Fasano *et al.,* 2016; Stanley *et al.,* 2016). Increased awareness of the efficacy of this transfusion modality both in the acute and chronic settings through cooperative projects such as the CDC (DD14‐1406) is indicated. Furthermore, recognising that this technology may not be cost‐effective for some institutions that care for small numbers of SCD patients, efficient centralisation of its use should be optimised.

Our study had certain strengths and limitations. The geographic bounds of the sample make it difficult to generalise findings nationally or internationally. Also, a potential bias stems from the variability in which an individual from each institution responded to the survey. Staff with different roles in a given blood bank may have different awareness of what practices are most typical there. Thus, some assessments of typical practice by individuals who responded to the survey might differ from what others in their blood bank would say; and some rejected answers from the hospitals from which we received multiple responses might better reflect usual practice at that institution than the retained ones. We were unable to follow up to resolve any intra‐institutional response discrepancies because we offered respondents anonymity to reduce social acceptability bias.

Although the number of responding institutions is relatively small, analysis of hospital discharge statistics from our data use agreement with Georgia Department of Public Health indicates that the Georgia hospitals in the survey that reported transfusing haemoglobinopathy patients represent approximately one‐third of all those that transfused such patients in 2016 (*n* = 87; infusion centres not included). Furthermore, these responding institutions accounted for 2037 (83%) of the 2458 transfusions given collectively to this population. Geographically, the 25 Georgia counties from which we received responses were distributed across the state, ranging from dense urban areas (metro Atlanta and suburbs) to sparsely populated rural locations (North Georgia mountain region; Central, South and coastal Georgia). Therefore, despite not having access to non‐responding institutions, we feel that the response sample is a good representation of institutions within Georgia that transfuse patients with haemoglobinopathies.

Despite these limitations, these data offer updated information on continued inconsistencies that exist in transfusion practices for patients with haemoglobinopathies despite NHLBI and TIF offering transfusion recommendations based on the best evidence available. Consequently, based on these inconsistencies in care along with inadequate intra‐institutional and inter‐institutional communication towards identifying these patients by transfusion services, alloimmunisation and DHTRs remain significant risks for patients with haemoglobinopathies.

## CONCLUSIONS

Inconsistencies exist in transfusion practices of SCD and TDT patients in non‐SCTC/non‐TTC and community‐based healthcare systems with regard to following NHLBI/TIF recommendations. This may be in part due to unfamiliarity with the recommendations, or a reluctance to adopt ‘cost‐adding’ transfusion practices without adequate high‐quality evidence, which supports a reduction in transfusion‐related outcomes. There is a potential need for expanding the role of advanced technologies in transfusion management, notably RBC genotyping and automated red cell exchange (particularly for SCD patients), through increased education and training, or centralising their use in resource‐limited geographic areas. In an expanding world of high‐quality, cost‐effective care, future support of transfusion‐based multi‐centre prospective randomised clinical trials along with implementation science research is critically needed to determine and promote the widespread adoption of evidence‐based practices in the transfusion management of patients with haemoglobinopathies.

## CONFLICT OF INTEREST

C. D. J. has functioned as a consultant for Octapharma, Immucor and Biomet Zimmer. R. M. F. has served as an advisory board member for Immucor, and gives educational talks for Terumo BCT. All authors have no relevant conflicts of interest.

## Supporting information


**Figure S1.** Blood bank survey.
**Table S1.** Response institution and respondent characteristics.
