# Supplementary material for: Transfusion service knowledge and immunohaematological practices related to sickle cell disease and thalassemia
Source: Transfus Med. 2019 Feb 10;29(3):185–92. doi: 10.1111/tme.12580 (PMC6767094; doi:10.1111/tme.12580)
Supplement: Supplementary file 1 — Figure S1. Blood bank survey. Table S1. Response institution and respondent characteristics. [file TME-29-185-s001.docx]

**Supplemental table 1**. Response institution and respondent characteristics

|  |  |  | Transfuse ≥1 SCD pt/month (n=32) | Transfuse ≥1  TDT pt/month  (n=11) |
| --- | --- | --- | --- | --- |
| Institution characteristics | Teaching hospitals |  | 19 (59%) | 7 (64%) |
|  | Designated trauma centers |  | 17 (53%) | 8 (73%) |
| Respondent's role in blood bank | Medical director |  | 3 (9%) | 1 (9%) |
|  | Transfusion service manager |  | 12 (38%) | 5 (45%) |
|  | Lab supervisor |  | 7 (22%) | 3 (27%) |
|  | Medical technologist |  | 10 (31%) | 2 (18%) |

Supplemental figure 1. Blood Bank survey

1 **Informed Consent** (not shown)

2 **Consent: If you agree to participate in this research, click CONTINUE to proceed with the survey.**

- CONTINUE
- EXIT

Skip To: End of Survey If Consent = EXIT

3 What best describes your title/role in the transfusion service?

- Medical director or assistant medical director
- Transfusion services manager
- Lab supervisor
- Medical technologist
- Other ___________________

Display This Question: If Role != Medical director or assistant medical director

4 Your certifications (check all that apply)

- Specialist in Blood Banking, SBB(ASCP)
- Technologist in Blood Banking, BB(ASCP)
- Pathologists' Assistant, PA(ASCP)
- Medical Assistant (RMA)
- Medical Technologist (MT)
- Medical Laboratory Technician (MLT)
- Medical Laboratory Assistant (CMLA)

Display This Question: If Role = Medical director or assistant medical director

5 Your medical training (check all that apply)

- Anatomic pathology/clinical pathology
- Clinical pathology
- Pediatric hematology/oncology
- Internal medicine - hematology/oncology
- Blood banking/transfusion medicine

6 Name of your institution (so we can combine responses from multiple staff members, if applicable) ___________

7 Is your institution a teaching hospital?

- No
- Yes

8 Is your institution a designated trauma center?

- No
- Yes, Level I
- Yes, Level II
- Yes, Level III
- Yes, Level IV

9 Is your institution affiliated with the following? (check all that apply)

- SEABB (Southeastern Area Blood Bankers)
- AABB (American Association of Blood Banks)
- CAP (College of American Pathologists)
- ASCP (American Society of Clinical Pathology)

10 In what county is your institution located?

| - Outside Georgia - Appling - Athens-Clarke - Atkinson - Augusta-Richmond - Bacon - Baker - Baldwin - Banks - Barrow - Bartow - Ben Hill - Berrien - Bibb - Bleckley - Brantley - Brooks - Bryan - Bulloch - Burke - Butts - Calhoun - Camden - Candler - Carroll - Catoosa - Charlton - Chatham - Chattooga - Cherokee - Clay - Clayton - Clinch - Cobb - Coffee - Colquitt - Columbia - Columbus-Muscogee | - Cook - Coweta Crawford - Crisp - Cusseta-Chattahoochee - Dade Dawson - Decatur - DeKalb - Dodge Dooly - Dougherty - Douglas - Early - Echols - Effingham Elbert - Emanuel - Evans - Fannin - Fayette - Floyd - Forsyth - Franklin - Fulton - Georgetown-Quitman - Calmer - Classics - Glynn - Gordon - Grady - Greene - Gwinnett - Habersham - Hall - Hancock - Haralson - Harris - Hart - Heard - Henry - Houston | - Irwin - Jackson - Jasper Jeff Davis - Jefferson - Jenkins - Johnson - Jones - Lamar - Lanier - Laurens Lee - Liberty - Lincoln - Long Lowndes - Lumpkin - Macon - Madison - Marion - McDuffie - McIntosh - Meriwether - Miller - Mitchell - Monroe - Montgomery Morgan - Murray - Newton - Oconee - Oglethorpe - Paulding - Peach - Pickens - Pierce - Pike - Polk - Pulaski - Putnam - Rabun - Randolph | - Rockdale - Schley - Screven - Seminole - Tift Spalding - Stephens - Stewart - Sumter - Talbot - Taliaferro - Tattnall - Taylor - Telfair - Terrell - Thomas - Toombs - Towns - Treutlen - Troup - Turner - Twiggs - Union - Upson - Walker - Walton - Ware - Warren - Washington - Wayne - Webster - Wheeler - White - Whitfield - Wilcox - Wilkes - Wilkinson - Worth |
| --- | --- | --- | --- |

11 About what percentage of your transfusion service's red blood cell (RBC) units are supplied by your own institution's blood donation center (as opposed to an external source like LifeSouth or American Red Cross)?

- 0%
- 10-40%
- 40-60%
- 60-90%
- >90%
- 100%

12 About how many total RBC units does your service issue in a year?

- 500-1,000
- 1,000-10,000
- 10,000-20,000
- >20,000

13 About how many patients with sickle cell disease (**SCD**) are transfused at your institution per month? (include patients with HbSS, HbSC, HbSβo-thalassemia and HbSβ+-thalassemia)

- 0
- 1-3
- 4-30
- 31-100
- >100

Display This Question: If SCpts != 0

14 About what portion of your transfused **SCD** patients are under age 18?

- 0%
- 10-40%
- 40-60%
- 60-90%
- >90%
- 100%

Display This Question: If SCpts != 0

15 What types of transfusion services does your institution provide for patients with **SCD**? (check all that apply)

- Simple transfusions
- Automated exchange transfusions
- Manual exchange transfusions
- Chronic transfusion program

Display This Question: If SCpts != 0

16 About how many of your **SCD** patients receive chronic transfusions? (chronic = 8 or more routinely scheduled transfusions in 12 months)

- 0
- 1-9
- 10-29
- 30-100
- >100

17 About how many patients with thalassemia major or thalassemia intermedia (**THAL**) are transfused at your institution per month? (exclude patients with sickle-thalassemia phenotypes, HbSβ0-thalassemia and HbSβ+-thalassemia)

- 0
- 1
- 2-5
- 6-30
- >30

Display This Question: If THpts != 0

18 What portion of your transfused **THAL** patients are under age 18?

- 0%
- 10-40%
- 40-60%
- 60-90%
- >90%
- 100%

Display This Question: If SCpts != 0 And THpts != 0

19 When transfusions are ordered for **SCD/THAL** patients, how likely are they to be ordered by each of the following?

|  | Very  Unlikely | Somewhat  Likely | Very  Likely |
| --- | --- | --- | --- |
| General pediatrician |  |  |  |
| Pediatric hematologist/oncologist or other sub-specialty |  |  |  |
| Adult internal medicine physician |  |  |  |
| Adult hematologist/oncologist or other sub-specialty |  |  |  |
| Emergency medicine physician |  |  |  |
| Surgeon |  |  |  |
| Hospitalist |  |  |  |
| Nurse practitioner or physician's assistant |  |  |  |
| Resident/Fellow |  |  |  |
|  |  |  |  |

Display This Question: If SCpts != 0

20 What requirements does your institution usually have for RBC units selected for transfusion to **SCD** patients? (check all that apply)

- Leukoreduced
- Irradiated
- Negative for sickle cell trait
- Less than __ days old (enter number of days) _________
- Don't know

Display This Question: If THpts != 0

21 What requirements does your institution usually have for RBC units selected for transfusion to **THAL** patients? (check all that apply)

- Leukoreduced
- Irradiated
- Negative for sickle cell trait
- Less than __ days old (enter number of days) _________
- Don't know

Display This Question: If SCpts != 0

22 Under non-emergency conditions, what is your institution’s usual procedure for determining the blood type/red cell phenotype of **SCD** patients who are not alloimmunized?

- Type for ABO/Rh(D)
- Type for ABO/Rh(D) and determine the baseline phenotype for Rh and Kell antigens
- Type for ABO/Rh(D) and determine the extended phenotype beyond Rh and Kell antigens

Display This Question: If SCtyp = Type for ABO/Rh(D) and determine the baseline phenotype for Rh and Kell antigens

23 Which antigens are included in the phenotype?

| - C - c - E - e | - K - k - Don't know |
| --- | --- |

Display This Question: If SCtyp = Type for ABO/Rh(D) and determine the extended phenotype beyond Rh and Kell antigens

24 Which antigens are included in extended phenotype?

| - C - c - E - e - K | - k - Fya - Fyb - Jka - Jkb | - M - N - S - s - Lua | - Lub - Dia Dib - Doa - Dob - Lea | - Leb - P - Other: _____ - Don't know |
| --- | --- | --- | --- | --- |

Display This Question: If THpts != 0

25 Under non-emergency conditions, what is your institution’s usual procedure for determining blood type/red cell phenotype for **THAL** patients who are not alloimmunized?

- Type for ABO/Rh(D)
- Type for ABO/Rh(D) and determine the baseline phenotype for Rh and Kell antigens
- Type for ABO/Rh(D) and determine the extended phenotype beyond Rh and Kell antigens

Display This Question: If THtyp = Type for ABO/Rh(D) and determine the baseline phenotype for Rh and Kell antigens

26 Which antigens are included in the phenotype?

| - C - c - E - e | - K - k - Don't know |
| --- | --- |

Display This Question: If THtyp = Type for ABO/Rh(D) and determine the extended phenotype beyond Rh and Kell antigens

27 Which antigens are included in extended phenotype?

| - C - c - E - e - K | - k - Fya - Fyb - Jka - Jkb | - M - N - S - s - Lua | - Lub - Dia Dib - Doa - Dob - Lea | - Leb - P - Other: _____ - Don't know |
| --- | --- | --- | --- | --- |

Display This Question: If SCpts != 0

28 Under non-emergency conditions, what is your institution’s usual procedure for selecting RBC units for transfusion to **SCD** patients with no current or past history of alloantibodies?

- Crossmatch for ABO/Rh(D) compatibility only
- Crossmatch for ABO/Rh(D) and C, c, E, e, K compatibility
- Crossmatch for ABO/Rh(D) compatibility plus extended matching including and beyond C, c, E, e, K

Display This Question: If SCmtch = Crossmatch for ABO/Rh(D) and C, c, E, e, K compatibility

29 Which antigens are included in the match?

| - C - c - E - e | - K - k - Don't know |
| --- | --- |

Display This Question: If SCmtch = Crossmatch for ABO/Rh(D) compatibility plus extended matching including and beyond C, c, E, e, K

30 Which antigens are included in extended match?

| - C - c - E - e - K | - k - Fya - Fyb - Jka - Jkb | - M - N - S - s - Lua | - Lub - Dia Dib - Doa - Dob - Lea | - Leb - P - Other: _____ - Don't know |
| --- | --- | --- | --- | --- |

Display This Question: If THpts != 0

31 Under non-emergency conditions, what is your institution’s usual procedure for selecting RBC units for transfusion to **THAL** patients?

- Crossmatch for ABO/Rh(D) compatibility only
- Crossmatch for ABO/Rh(D) and C, c, E, e, K compatibility
- Crossmatch for ABO/Rh(D) compatibility plus extended matching including and beyond C, c, E, e, K

Display This Question: If THmtch = Crossmatch for ABO/Rh(D) and C, c, E, e, K compatibility

32 Which antigens are included in the match?

| - C - c - E - e | - K - k - Don't know |
| --- | --- |

Display This Question: If THmtch = Crossmatch for ABO/Rh(D) compatibility plus extended matching including and beyond C, c, E, e, K

33 Which antigens are included in extended match?

| - C - c - E - e - K | - k - Fya - Fyb - Jka - Jkb | - M - N - S - s - Lua | - Lub - Dia Dib - Doa - Dob - Lea | - Leb - P - Other: _____ - Don't know |
| --- | --- | --- | --- | --- |

Display This Question: If SCpts != 0 Or THpts != 0

34 Does your institution try to select RBC units that have been stored in a particular solution for transfusion to **SCD/THAL** patients?

- No
- Yes, CPDA storage preferred (citrate phosphate dextrose adenine)
- Yes, extended storage anticoagulant-preservative solutions preferred (i.e. AS-1, AS-3)

Display This Question: If SCpts != 0

35 Under non-emergency conditions, which of the following best describes your institution’s usual procedure for selecting donor RBC units for transfusion to **SCD** patients with a history of a single alloantibody to a clinically significant RBC antigen (e.g., anti-Jkb)?

- ABO/Rh(D) compatible (not matched for antigens beyond ABO and D)
- ABO/Rh(D) compatible AND negative for the antigen(s) to which the patient has antibody(s)
- ABO/Rh(D) compatible and limited match (C, c, E, e, K) and negative for the antigen(s) to which the patient has antibody(s)
- ABO/Rh(D) compatible and extended match beyond C, c, E, e, K and negative for the antigen(s) to which the patient has antibody(s)

Display This Question: If SC1ab = ABO/Rh(D) compatible and limited match (C, c, E, e, K) and negative for the antigen(s) to which the patient has antibody(s)

36 Which antigens are included in the match?

| - C - c - E - e | - K - k - Don't know |
| --- | --- |

Display This Question: If SC1ab = ABO/Rh(D) compatible and extended match beyond C, c, E, e, K and negative for the antigen(s) to which the patient has antibody(s)

37 Which antigens are included in extended match?

| - C - c - E - e - K | - k - Fya - Fyb - Jka - Jkb | - M - N - S - s - Lua | - Lub - Dia Dib - Doa - Dob - Lea | - Leb - P - Other: _____ - Don't know |
| --- | --- | --- | --- | --- |

Display This Question: If THpts != 0

38 Under non-emergency conditions, which of the following best describes your institution’s usual procedure for selecting donor RBC units for transfusion to **THAL** patients with a history of a single alloantibody to a clinically significant RBC antigen (e.g., anti-Jkb)?

- ABO/Rh(D) compatible (not matched for antigens beyond ABO and D)
- ABO/Rh(D) compatible AND negative for the antigen(s) to which the patient has antibody(s)
- ABO/Rh(D) compatible and limited match (C, c, E, e, K) and negative for the antigen(s) to which the patient has antibody(s)
- ABO/Rh(D) compatible and extended match beyond C, c, E, e, K and negative for the antigen(s) to which the patient has antibody(s)

Display This Question: If TH1ab = ABO/Rh(D) compatible and limited match (C, c, E, e, K) and negative for the antigen(s) to which the patient has antibody(s)

39 Which antigens are included in the match?

| - C - c - E - e | - K - k - Don't know |
| --- | --- |

Display This Question: If TH1ab = ABO/Rh(D) compatible and extended match beyond C, c, E, e, K and negative for the antigen(s) to which the patient has antibody(s)

40 Which antigens are included in extended match?

| - C - c - E - e - K | - k - Fya - Fyb - Jka - Jkb | - M - N - S - s - Lua | - Lub - Dia Dib - Doa - Dob - Lea | - Leb - P - Other: _____ - Don't know |
| --- | --- | --- | --- | --- |

Display This Question: If SCpts != 0

41 Under non-emergency conditions, which of the following best describes your institution’s usual procedure for selecting donor RBC units for transfusion to **SCD** patients with a history of 2 or more alloantibodies to clinically significant RBC antigens (e.g., anti-Jkb and anti-Fya)?

- ABO/Rh(D) compatible (not matched for antigens beyond ABO and D)
- ABO/Rh(D) compatible AND negative for the antigen(s) to which the patient has antibody(s)
- ABO/Rh(D) compatible and limited match (C, c, E, e, K) and negative for the antigen(s) to which the patient has antibody(s)
- ABO/Rh(D) compatible and extended match beyond C, c, E, e, K and negative for the antigen(s) to which the patient has antibody(s)

Display This Question: If SC2ab = ABO/Rh(D) compatible and limited match (C, c, E, e, K) and negative for the antigen(s) to which the patient has antibody(s)

42 Which antigens are included in the match?

| - C - c - E - e | - K - k - Don't know |
| --- | --- |

Display This Question: If SC2ab = ABO/Rh(D) compatible and extended match beyond C, c, E, e, K and negative for the antigen(s) to which the patient has antibody(s)

43 Which antigens are included in extended match?

| - C - c - E - e - K | - k - Fya - Fyb - Jka - Jkb | - M - N - S - s - Lua | - Lub - Dia Dib - Doa - Dob - Lea | - Leb - P - Other: _____ - Don't know |
| --- | --- | --- | --- | --- |

Display This Question: If THpts != 0

44 Under non-emergency conditions, which of the following best describes your institution’s usual procedure for selecting donor RBC units for transfusion to **THAL** patients with a history of 2 or more alloantibodies to clinically significant RBC antigens (e.g., anti-Jkb and anti-Fya)?

- ABO/Rh(D) compatible (not matched for antigens beyond ABO and D)
- ABO/Rh(D) compatible AND negative for the antigen(s) to which the patient has antibody(s)
- ABO/Rh(D) compatible and limited match (C, c, E, e, K) and negative for the antigen(s) to which the patient has antibody(s)
- ABO/Rh(D) compatible and extended match beyond C, c, E, e, K and negative for the antigen(s) to which the patient has antibody(s)

Display This Question: If TH2ab = ABO/Rh(D) compatible and limited match (C, c, E, e, K) and negative for the antigen(s) to which the patient has antibody(s)

45 Which antigens are included in the match?

| - C - c - E - e | - K - k - Don't know |
| --- | --- |

Display This Question: If TH2ab = ABO/Rh(D) compatible and extended match beyond C, c, E, e, K and negative for the antigen(s) to which the patient has antibody(s)

46 Which antigens are included in extended match?

| - C - c - E - e - K | - k - Fya - Fyb - Jka - Jkb | - M - N - S - s - Lua | - Lub - Dia Dib - Doa - Dob - Lea | - Leb - P - Other: _____ - Don't know |
| --- | --- | --- | --- | --- |

Display This Question: If SCpts != 0

47 How does your transfusion service know if a new patient to be transfused has **SCD**? (check all that apply)

- Through a crossmatch order with “sickle-negative” restriction requested
- By the “admission diagnosis” identified in the medical record
- Through a required Q/A on the type/screen or crossmatch order asking if the patient has SCD
- Through verbal communication from the ordering physician
- We have no routine system for finding out that a new patient has SCD.
- Other ______________________

Display This Question: If SCpts != 0

48 Under non-emergency conditions, which of the following best describes your institution’s usual procedures for minimizing risk of delayed hemolytic transfusion reactions (DHTRs) in patients with **SCD**? (check all that apply)

- We depend on the patients’ clinical provider to obtain a transfusion history to identify whether transfusions have occurred at other institutions (clinical provider-initiated). If there is a history of transfusions

elsewhere, we contact the institution(s) where the patient was transfused to obtain antibody history prior to cross-matching units.

- We contact the provider who orders a type/cross on a new SCD patient to obtain a transfusion history. If there is a history of transfusions elsewhere, we then contact the institution(s) where the patient was transfused to obtain antibody history prior to cross-matching units.
- We contact the provider ordering a type/cross on a SCD patient if they have not had a transfusion at our institution in over X years (please specify below) to obtain an interim transfusion history. If there is a history of

transfusions elsewhere, we then contact the institution(s) where patient was transfused to obtain antibody history prior to cross-matching units. _______________

- We have no routine system for identifying SCD patients who may have been transfused elsewhere.
- Other ______________________
- Don't know

Display This Question: If SCpts != 0

49 Which of the following best describes your institution’s usual procedure for evaluating a **SCD** patient with a positive antibody screen with anti-e specificity and a negative DAT, with the following phenotype:  D+, C-, E-, c+, e+, K-, k+, Fya-, Fyb- , Jka+, Jkb-, M+, N+, S-, s+ ?

- Consider this an autoantibody and provide “least incompatible” e-positive RBC units
- Consider this an autoantibody and provide “least incompatible” e-negative (DcE/DcE) RBC units
- Consider this may be a possible alloantibody to Rh e in a patient with an Rh variant and provide "least incompatible" e-positive (i.e. Dce/Dce) RBC units.
- Consider this may be a possible alloantibody to Rh e in a patient with an Rh variant and provide e-negative (DcE/DcE) RBC units
- Consider this may be a possible alloantibody to Rh e in a patient with an Rh variant and perform red cell genotyping to evaluate for presence of RH variant haplotypes. If genotyping confirms the presence of an Rh variant which predicts loss of a high-incidence Rh antigen (e.g. hrB-), provide e-negative (DcE/DcE) RBC units.
- Consider this may be a possible alloantibody to Rh e in a patient with an Rh variant and perform red cell genotyping to evaluate for presence of RH variant haplotypes. If genotyping confirms the presence of an Rh

variant which predicts loss of a high-incidence Rh antigen (e.g. hrB-), provide genotypic-match RBC units or RBC units lacking the high-incidence Rh antigen (hrB- units).

- Other _______________________
- Don't know

Display This Question: If SCpts != 0

50 What clinical scenarios would usually trigger a request for physical transfer of an **SCD** patient to a specialized center? (check all that apply)

- Need for red cell exchange (erythrocytapheresis)
- Development of multiple RBC alloantibodies (enter how many) __________________________________
- Development of complicated RBC alloantibodies (Please define: anti-e in an e+ pt, Abs to high prevalence antigens i.e. Jsb, HTLA antibodies) ________________________________
- Development of autoantibodies
- Need for extended match units (beyond CEK)
- N/A: We are a referral center

Display This Question: If SCpts != 0

51 Does your transfusion service use red cell molecular phenotyping (red cell genotyping) for evaluating red cell phenotype in **SCD** patients?

- No, we do not use molecular phenotyping.
- Yes, we use it for for all SCD patients.
- Yes, we use it when the patient's phenotype is not available and they have been transfused within the past 3 months.
- Yes, but only for patients with complicated red cell antibody reactivity. (Please describe examples.) ______
- Yes; other. (Please explain.) ____________________

Display This Question: If SCpts != 0 Or THpts != 0

52 What are the main challenges you face in transfusion management of **SCD/THAL** patients? _________________

53 What educational/training content would be most helpful to your transfusion service staff? ________________

54 How effective are these methods for informing you about guidelines and best practices in transfusion?

| Method | Not effective | Minimally effective | Moderately effective | Highly effective |
| --- | --- | --- | --- | --- |
| Email |  |  |  |  |
| Surface mail |  |  |  |  |
| Live CME |  |  |  |  |
| Webinars |  |  |  |  |
| Mobile apps (Android and iPhone compatible) |  |  |  |  |
| Professional association newsletters/listservs (list) |  |  |  |  |
| Scientific journals (list) |  |  |  |  |
| Other methods (list) |  |  |  |  |
